# Absence of endogenous carnosine synthesis does not increase protein carbonylation and advanced lipoxidation end products in brain, kidney or muscle

**DOI:** 10.1007/s00726-022-03150-8

**Published:** 2022-03-16

**Authors:** Lihua Wang-Eckhardt, Ivonne Becker, Yong Wang, Jing Yuan, Matthias Eckhardt

**Affiliations:** 1 Institute of Biochemistry and Molecular Biology, Medical Faculty, University of Bonn, Nussallee 11, 53115 Bonn, Germany; 2 Present Address: Shandong Xinchuang Biotechnology Co., LTD, Jinan, China; 3 Present Address: College of Animal Science, Yangtze University, Jingzhou, Hubei China

**Keywords:** Advanced lipoxidation end products, Carnosine, Diabetes, Protein carbonylation

## Abstract

Carnosine and other histidine-containing dipeptides are expected to be important anti-oxidants in vertebrates based on various in vitro and in vivo studies with exogenously administered carnosine or its precursor β-alanine. To examine a possible anti-oxidant role of endogenous carnosine, mice lacking carnosine synthase (Carns1^−/−^) had been generated and were examined further in the present study. Protein carbonylation increased significantly between old (18 months) and aged (24 months) mice in brain and kidney but this was independent of the Carns1 genotype. Lipoxidation end products were not increased in 18-month-old Carns1^−/−^ mice compared to controls. We also found no evidence for compensatory increase of anti-oxidant enzymes in Carns1^−/−^ mice. To explore the effect of carnosine deficiency in a mouse model known to suffer from increased oxidative stress, Carns1 also was deleted in the type II diabetes model Lepr^db/db^ mouse. In line with previous studies, malondialdehyde adducts were elevated in Lepr^db/db^ mouse kidney, but there was no further increase by additional deficiency in Carns1. Furthermore, Lepr^db/db^ mice lacking Carns1 were indistinguishable from conventional Lepr^db/db^ mice with respect to fasting blood glucose and insulin levels. Taken together, Carns1 deficiency appears not to reinforce oxidative stress in old mice and there was no evidence for a compensatory upregulation of anti-oxidant enzymes. We conclude that the significance of the anti-oxidant activity of endogenously synthesized HCDs is limited in mice, suggesting that other functions of HCDs play a more important role.

**Supplementary Information:**

The online version contains supplementary material available at 10.1007/s00726-022-03150-8.

## Introduction

The dipeptide carnosine (β-alanyl-l-histidine) and related histidine-containing dipeptides (HCDs) have been found in all mammals analysed and in many vertebrates (Boldyrev et al. 2013). Carnosine is synthesized by an amino acid ligase, carnosine synthase, encoded by the carnosine synthase 1 gene (Carns1; EC 6.3.2.11), also known as ATP-grasp domain containing 1 gene (Drozak et al. 2010; Kwiatkowski et al. 2018). By far the highest concentrations of HCDs (mM range) in mammals are present in skeletal muscle (Gulewitsch and Amiradzibi 1900; Boldyrev et al. 2013) and receptor neurons of the olfactory epithelium (Margolis, 1974). Mammalian heart muscle also contains high concentration (mM range) of HCDs, though mainly as *N*-acetylated derivatives (O'Dowd et al. 1988). Much lower HCD concentrations are found in other tissues, with relatively high levels (upper µM range in mice) in brain and kidney (Boldyrev et al. 2013). Carnosine is degraded by cytosolic carnosinase CN2 (EC 3.4.13.18) and serum carnosinase CN1 (EC 3.4.13.20).

Numerous roles have been proposed for carnosine and other HCDs (Boldyrev et al. 2013). Carnosine has been shown to act as an anti-oxidant (Boldyrev et al. 1987; Kohen et al. 1988) and to inhibit the formation of advanced glycation and lipoxidation end products (Boldyrev et al. 2013). An anti-diabetic activity of carnosine has been documented in different animal models of type II diabetes (Sauerhöfer et al. 2007; Albrecht et al. 2017; Peters et al. 2018a; Artioli et al. 2019). A polymorphism of the CNDP1 gene resulting in a CN1 serum carnosinase with lower enzymatic activity is associated with reduced susceptibility for nephropathy in type II diabetes (Janssen et al. 2005). In line with this, carnosinase overexpression aggravates diabetes in diabetic mouse models (Sauerhöfer et al. 2007; Everaert et al. 2020; Qiu et al. 2020).

Carnosine deficient (Carns1^−/−^) mice (Wang-Eckhardt et al. 2020) and rats (de Souza Gonçalves et al. 2021) have been generated to further examine the physiological role of endogenous HCDs. These studies provided clear evidence for a role of carnosine in the maintenance of the olfactory epithelium in old and aging mice (Wang-Eckhardt et al. 2020) and in excitation–contraction coupling in rat cardiomyocytes (de Souza Gonçalves et al. 2021). In contrast, both studies could not identify an important role of carnosine and anserine in skeletal muscle. The present study was undertaken to further examine whether absence of endogenous carnosine synthesis increases protein oxidation and lipoxidation in old and aging mice, as suggested by the anti-oxidant function of administered carnosine. We focused on tissues with the highest HCD concentrations, i.e. skeletal muscle, heart, brain and kidney (Boldyrev et al. 2013).

## Materials and methods

### Carns1^−/−^ and Lepr^db/db^ mice and genotyping

Carns1^−/−^ mice have been described previously (Wang-Eckhardt et al. 2020). Leptin receptor mutant Lepr^db/+^ mice (Coleman 1978) were obtained from Jackson Laboratories (stock number 000697). Carns1^−/−^ mice were crossed with Lepr^db/+^ mice to obtain double deficient Lepr^db/db^/Carns1^−/−^ mice. Genotyping of Carns1 was done as described (Wang-Eckhardt et al. 2020). Lepr genotyping was done by PCR using oligonucleotides 5′-AGA ACG GAC ACT CTT TGA AGT CTC-3′ and 5′-CAT TCA AAC CAT AGT TTA GGT TTG TGT-3′ followed by digestion with the restriction endonuclease *Rsa*I, resulting in two fragments (108 bp and 27 bp) for the db allele and a 135 bp undigested PCR product for the wild-type allele. In the study using the Carns1 line, only males or females were compared in the individual experiments. However, for the quantitative analysis of all experiments data from males and females were combined. Forebrains were used for the analysis of brain tissues and musculus gastrocnemius for skeletal muscle.

### Western blot analysis

Tissues were homogenized in 50 mM sodium phosphate (pH 7.4) with freshly added protease inhibitors (cOmplete protease inhibitor mix from Roche, Basel, Switzerland; or 1 × HALT from Thermo Fisher, Waltham, Massachusetts, USA). Protein concentrations were measured using the Biorad DC protein assay with bovine serum albumin as standard. SDS-PAGE (8%, 10% or 12.5% acrylamide, as indicated in the Figure legends) of proteins (10 or 20 µg/lane) was performed using acrylamide solution with a acrylamide:bisacrylamide ratio of 29:1 (cat# A515, Carl Roth, Karlsruhe, Germany) and the Mini-Protean Tetra cell gel electrophoresis system (Biorad, Hercules, California, USA), as described (Gallagher 2012). Semi-dry Western blot transfer onto nitrocellulose membranes (Amersham Protran 0.1 µm NC, cat# 10600000, GE Healthcare, Chicago, Illinois, USA) was performed using a Trans-Blot SD Semi-Dry Transfer Cell (Biorad) and standard procedures (Goldman et al. 2015). Protein marker used was PageRuler Prestained Protein Ladder (cat# 26617, Lot# 00395064, Thermo Fisher). Peroxidase labelled secondary antibodies were detected using Pierce ECL Western blotting substrate (Thermo Fisher) and a CCD camera (Fusion Solo with FusionCapt Advance Solo 4 software; Vilber Lourmat, Eberhardzell, Germany) and images were saved as TIFF files. Primary antibodies used: rabbit anti-Carns1 (Wang-Eckhardt et al. 2020; dilution 1:1000), rabbit anti-HNE (HNE11, Alpha Diagnostics, Reinach, Switzerland, Lot# 301718S31-P; dilution 1:4000), rabbit anti-MDA (MDA11, Alpha Diagnostics, Lot# 29111259-P; dilution 1:4000), mouse anti-GSR (sc-133245, Santa Cruz Biotechnology, Dallas, Texas, USA, Lot# C0519; dilution 1:2000), mouse anti-SOD1 (sc-101523, Santa Cruz, Lot# I0418; dilution 1:2000), mouse anti-SOD2 (sc-133134, Santa Cruz, Lot# A1520; dilution 1:2000), mouse anti-actin (A5316, Sigma-Aldrich, St. Louis, Missouri, USA, Lot# 018M4804V; dilution 1:10000), rabbit anti-α-tubulin (600-401-880, Rockland, Limerick, Pennsylvania, USA; dilution 1:20000). Secondary antibodies used: goat anti-rabbit peroxidase (111-035-003, Dianova, Hamburg, Germany; dilution 1:20000), goat anti-mouse peroxidase (115-035-044, Dianova; dilution 1:20000). Signal intensities of whole lanes or individual protein bands were determined by densitometry using the program AIDA image analysis software (Elysia-raytest, Straubenhardt, Germany) and TIFF files of Western blot images with signal intensities in the linear range.

### Measurements in blood samples

All measurements in blood or plasma were performed using blood samples collected from the heart after mice have been killed by cervical dislocation.

Measurement of glycated haemoglobin: blood samples were mixed with four volumes of haemolysis puffer (150 mM NH_4_Cl, 1 mM KHCO_3_, 0.1 mM EDTA, pH 7.4) for five min, centrifuged at 3000×*g* for 10 min, supernatants removed and stored at -80 °C. Haemolysates were diluted in PBS and subjected to mouse GHbA1c ELISA (cat# E0709Mo; Bioassay Technology Laboratory, Shanghai, China) to determine glycated haemoglobin. Total haemoglobin was determined spectrophotometrically at 546 nm and the percentage of glycated haemoglobin was calculated.

Blood glucose determination: Fasting blood glucose (FBG) levels were determined after an overnight fasting period of 18 h using a blood glucose meter (Accu-Chek Aviva, Roche).

Plasma insulin determination: Plasma insulin was measured by ELISA (Ultra Sensitive Mouse Insulin ELISA Kit, cat# 90,080, Lot#09MYUMI081, Crystal Chem, Elk Grove Village, Iillinois, USA), following the manufacturer's instruction.

### Quantification of protein carbonyl content

Protein carbonyl content in tissue homogenates (brain, kidney, skeletal muscle) was determined spectrophotometrically as described (Reznick and Packer 1994). Briefly, tissues were homogenized in 50 mM sodium phosphate (pH 7.4), containing complete protease inhibitor cocktail (Roche) and the supernatant (1 mL) obtained after centrifugation for 10 min at 6000×*g* was incubated with 10 mM 2,4-dinitrophenylhydrazine (DNPH) in 2.5 M HCl (4 mL) or only 2.5 M HCl (control) for one hour at room temperature (light protected). After precipitation with one volume of 20% (w/v) TCA, washing with 10% TCA and 3-times with ethanol/ethyl acetate (1:1, v/v), the protein pellet was dissolved in 6 M guanidine hydrochloride and absorbance was measured at 360 nm. Dinitrophenyl (DNP) content was calculated using an absorbance coefficient of 22,000 M^−1^ cm^−1^ (Reznick and Packer 1994). Protein content of homogenates was determined using DC protein assay (Bio-Rad). Western blot analysis of carbonylated proteins was done as described (Levine et al. 1994). Briefly, homogenates were mixed with one volume of 12% SDS, followed by two volumes of 20 mM DNPH in 10% trifluoroacetic acid (TFA) or 10% TFA for controls. After one hour reaction time, samples were neutralized by addition of 2 M Tris/30% glycerol and analysed by SDS-PAGE and Western blotting using rabbit anti-DNP antiserum (cat# D9656, Sigma-Aldrich, Lot# 110M4801; dilution 1:5000). Signal intensities were determined as described above in the Western blot analysis section.

### Statistics

Data are shown as the mean ± standard deviation (SD) of at least three independent experiments (*n* = number of mice examined). Data were analysed using Excel (Microsoft, Richmond, Virginia, USA) or STATISTICA 6.0 (Statsoft, Tulsa, Oklahoma, USA). Data were tested for significant differences (*p* < 0.05) using one-way or two-way ANOVA with post hoc Tukey HSD test or two-tailed Student's *t* test.

## Results

### Carns1^−/−^ mice do not have elevated levels of carbonylated proteins

**Fig. 1:**
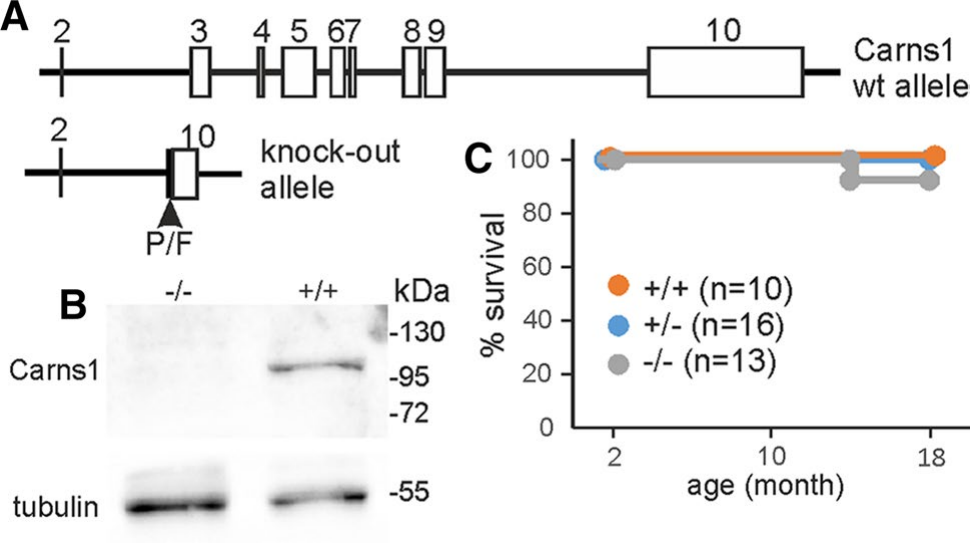
Carns1^−/−^ mice. **A** Schematic presentation of the wild type Carns1 gene and the Carns1 knock-out allele in Carns1^−/−^ mice analysed in this study. P/F indicate position of loxP and FRT sites that remain after cre mediated recombination in the knockout allele. All coding exons are deleted. **B** Western blot analysis (10% SDS-PAGE; 20 µg protein/lane) of skeletal muscle confirmed absence of Carns1 protein in Carns1^−/−^ mice. **C** Kaplan–Meier curve showed no significant difference between genotypes in survival rate

**Fig. 2:**
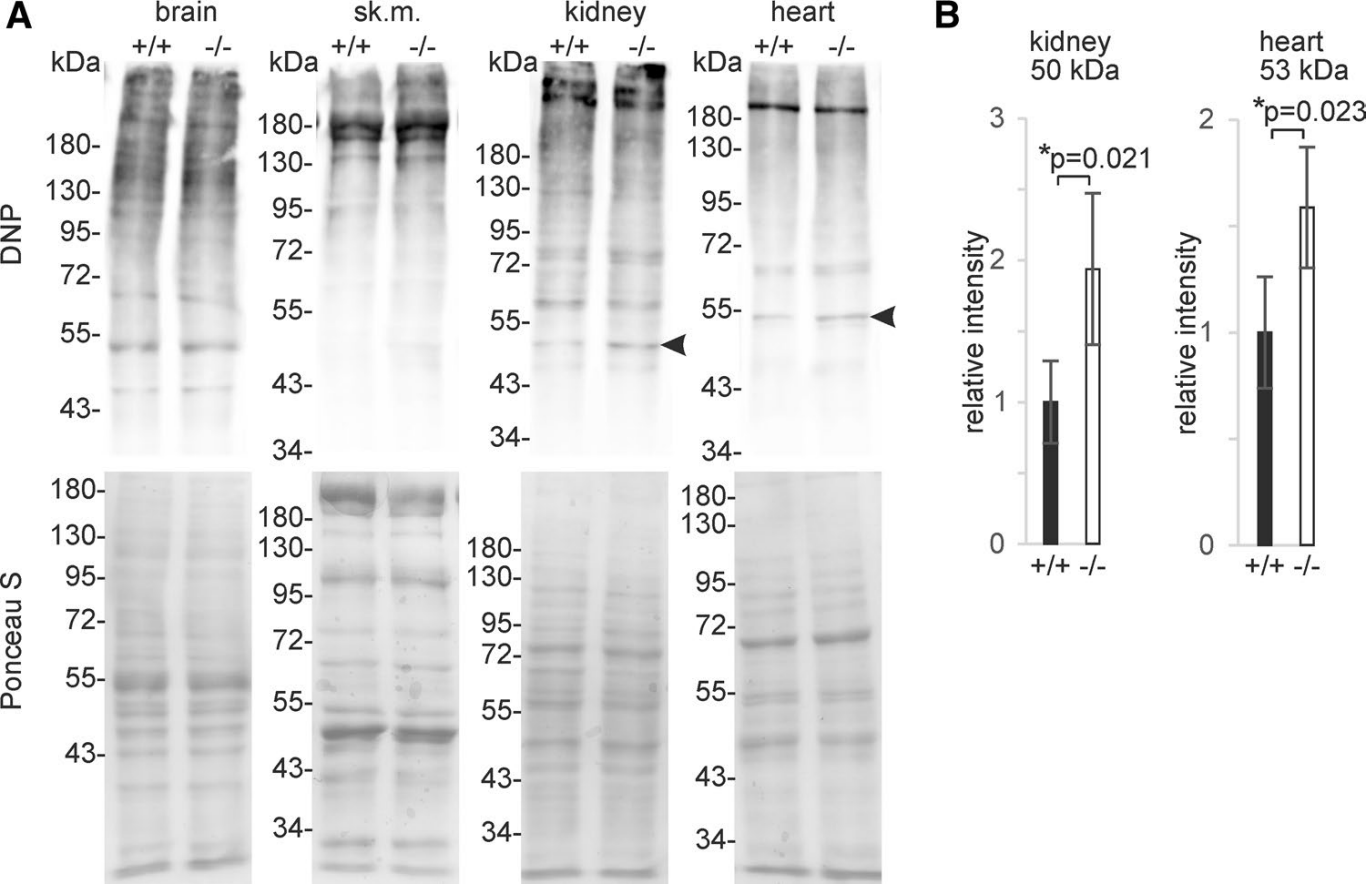
Protein carbonylation in brain, skeletal muscle, kidney and heart from Carns1^+/+^ and Carns1^−/−^ mice. **A** Tissue homogenates were treated with DNPH and analysed by 8% SDS-PAGE (10 µg protein/lane) and Western blotting using DNP specific antiserum. Age of mice was 18 months. Ponceau S staining served as loading control. In total, samples from at least 4 mice per genotype were examined. Representative experiments are shown. **B** Densitometric analysis showed that protein carbonyl bands at 50 kDa in kidney and 53 kDa in heart (labeled by arrow heads in A) had higher intensity in Carns1^−/−^ mice. Data shown are mean ± SD (*n* = 4 mice per genotype). Quantification data for other protein bands are given in supplementary Fig. S1

Carns1^−/−^ mice used in this study lacked exons 3 to 9 and part of exon 10, i.e. all parts of the gene encoding amino acids of the Carns1 protein (Fig. 1A, B. We focused our analysis on old mice, as we expected it more likely that carnosine deficiency becomes noticeable in aging mice that are more prone to oxidative stress. A Kaplan–Meier plot showed normal survival rate of mice used for experiments of 18-month-old mice (Fig. 1C). Levels of protein-carbonylation in brain, skeletal muscle, kidney and heart were determined in 18-month-old mice by Western blot analysis after derivatization with DNPH (Fig. 2). Densitometric quantification of individual bands (see supplementary Figure S1) revealed higher intensities of single bands at about 50 kDa in kidney and 53 kDa in heart from Carns1^−/−^ mice (arrow head in Fig. 2). We were, however, unable to decide whether this was caused by stronger carbonylation or increase in the concentration of a specific protein. There was an apparent tendency towards higher carbonyl content in skeletal muscle from Carns1^−/−^ mice, however, we could not detect significantly increased DNP levels by spectrophotometry in skeletal muscle, as well as in brain and kidney (Table 1). Because of a low signal-to-noise ratio, we were unable to obtain reliable data for heart tissue by spectrophotometry; the Western blot analysis, however, suggested similar total DNP levels in both genotypes, besides the increase of protein carbonylation at 53 kDa (Fig. 2). In 24-month-old mice protein carbonyl content increased in brain (two-way ANOVA, effect of age: *F*_1,11_ = 55.18, *p* < 0.0001) and kidney (effect of age: *F*_1,11_ = 24.41; *p* < 0.001) compared to 18-month, but there was no significant effect of genotype (Table 1). Thus, we found no evidence that endogenous carnosine protects from increasing protein carbonylation in aging mice.

**Table 1:** Protein carbonyl content in brain, kidney and skeletal muscle

Tissue	Age (months)	Carns1^+/+^	Carns1^−/−^	Two-way ANOVA
Brain	18	1.14 ± 0.22 (*n* = 3)	1.10 ± 0.19 (*n* = 3)	Effect of age (*p* < 0.001)
	24	1.93 ± 0.22 (*n* = 4)	1.82 ± 0.16 (*n* = 4)	
Kidney	18	0.76 ± 0.14 (*n* = 3)	0.98 ± 0.49 (*n* = 3)	Effect of age (*p* < 0.001)
	24	1.66 ± 0.36 (*n* = 4)	1.76 ± 0.24 (*n* = 4)	
Skeletal muscle	18	1.60 ± 0.63 (*n* = 6)	2.18 ± 0.42 (*n* = 6)	No significant effect
	24	1.62 ± 0.19 (*n* = 4)	1.79 ± 0.15 (*n* = 4)	

Carbonyl content (nmol/mg protein) in the indicated tissues from 18- and 24-month-old mice was determined spectrophotometrically after derivatization with DNPH, as described in “Materials and methods” (shown are mean ± SD; *n* = 3–6)

### Carns1^−/−^ mice do not have elevated levels of advanced lipoxidation end products

**Fig. 3:**
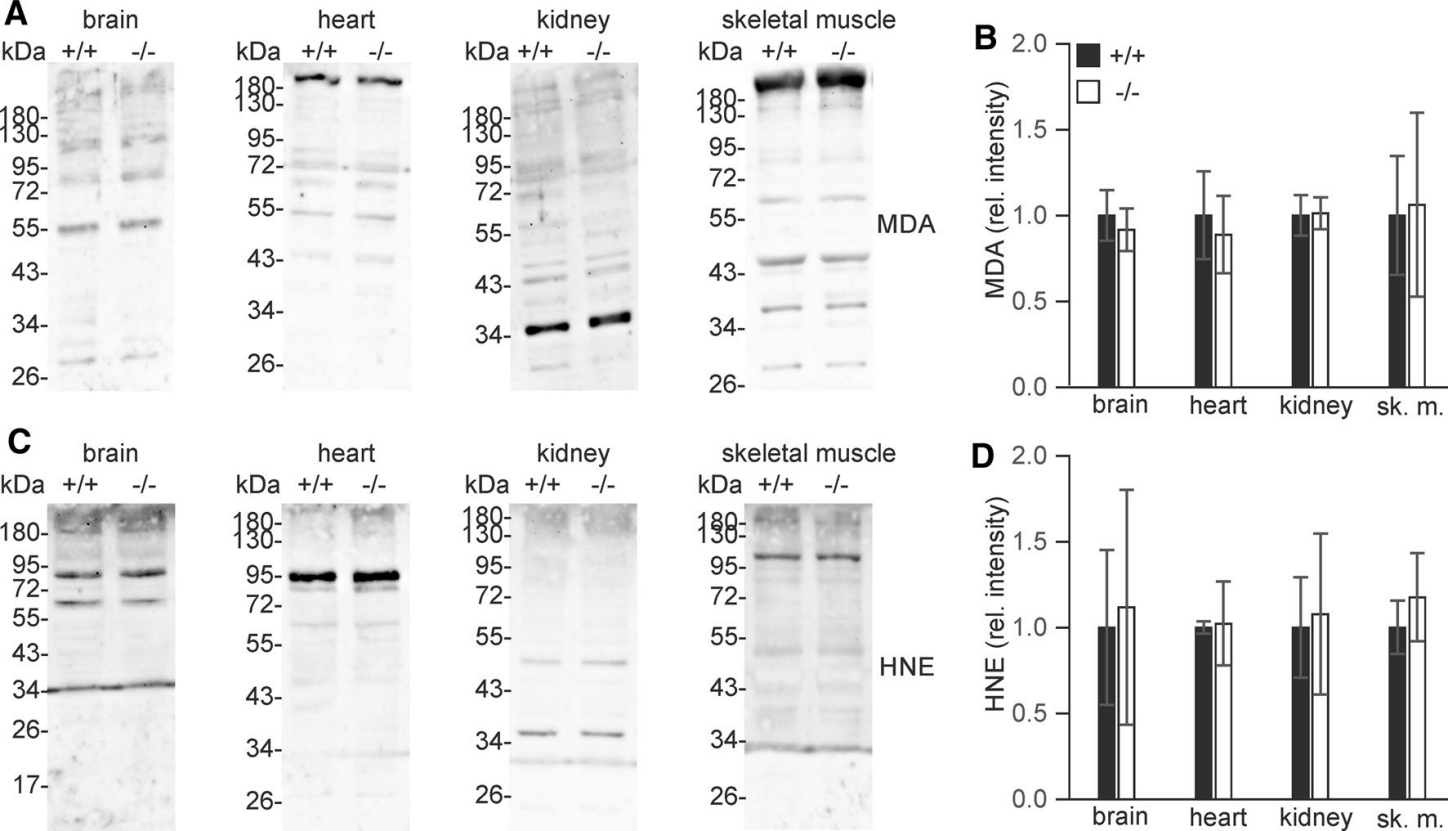
Western blot analysis of MDA and HNE adducts in brain, kidney, heart and skeletal muscle from 18-month-old mice. Proteins (20 µg/lane) were separated by 10% SDS-PAGE. **A** MDA Western blots. **B** Quantification of MDA Western blots. **C** HNE Western blots. **D** Quantification of HNE Western blots. Shown are the mean ± SD; mean of Carns1^+/+^ set to 1 (*n* = 4 mice per genotype). Quantification data for individual protein bands are shown in supplementary Fig. S2 and S3

To investigate to what extent endogenous carnosine may be essential for quenching lipid peroxidation products, levels of malondialdehyde (MDA) and 4-hydroxynonenal (HNE) protein adducts in brain, skeletal muscle, kidney, and heart were determined (Fig. 3). Densitometry of Western blots showed that there was neither a significant increase of individual MDA or HNE adduct bands (see supplementary Figs. S2 and S3) nor of the total amount of these adducts in Carns1^−/−^ mice in any of the tissues examined. Additional experiments were done with younger mice (age of 7–8 months), which also showed no significant differences between genotypes (data not shown). We conclude that lipoxidation products were not increased in the absence of endogenous carnosine and related peptides, suggesting that there was no significant increase of oxidative stress in Carns1^−/−^ mice.

### Anti-oxidant enzymes are not upregulated in Carns1^−/−^ mice

**Fig. 4:**
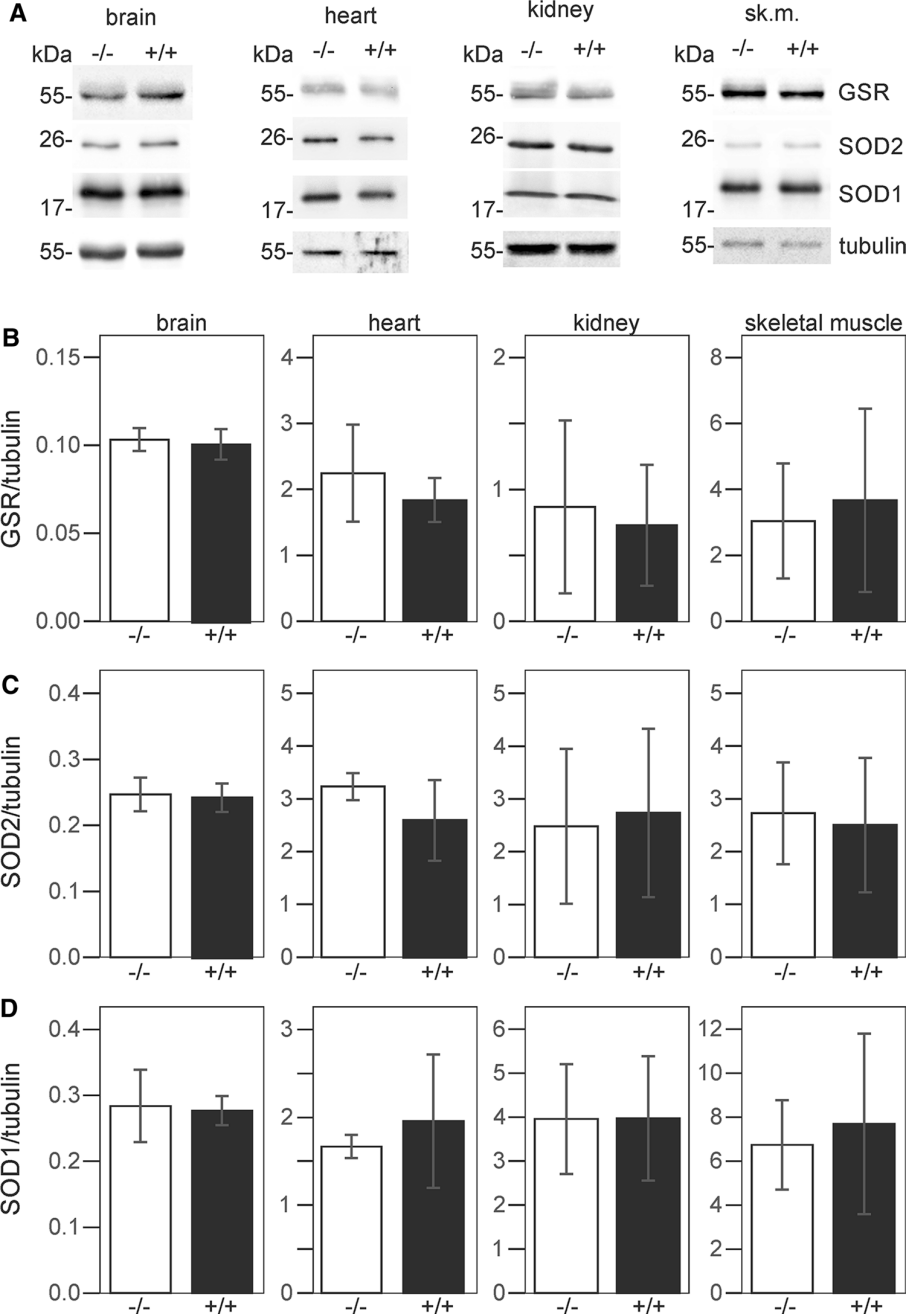
Western blot analysis of anti-oxidant enzymes. Proteins (10 µg/lane) were separated by 12.5% SDS-PAGE. **A** Representative Western blots of brain, heart, kidney and skeletal muscle from 18-month-old Carns1^+/+^ and Carns1^−/−^ mice are shown. Blots were stained with antibodies against GSR, SOD1, SOD2 and tubulin. **B** Densitometric quantification of Western blots (normalized to tubulin). No significant differences were observed. Shown are the mean ± SD (*n* = 4 mice per genotype)

Absence of elevated oxidative stress in Carns1^−/−^ mice could be the result of a compensatory upregulation of anti-oxidant enzymes, e.g. superoxide dismutase, in response to oxidative stress (Miao and St. Clair 2009). We therefore examined levels of three anti-oxidant enzymes: glutathione reductase (GSR), superoxide dismutase 1 (SOD1, CuZnSOD) and superoxide dismutase 2 (SOD2, MnSOD) in brain, heart, kidney, and skeletal muscle (Fig. 4A). Mean levels of the three enzymes were not significantly altered in all tissues examined.

### Absence of carnosine does not reinforce lipoxidation in kidney of Lepr^db/db^ mice

**Fig. 5:**
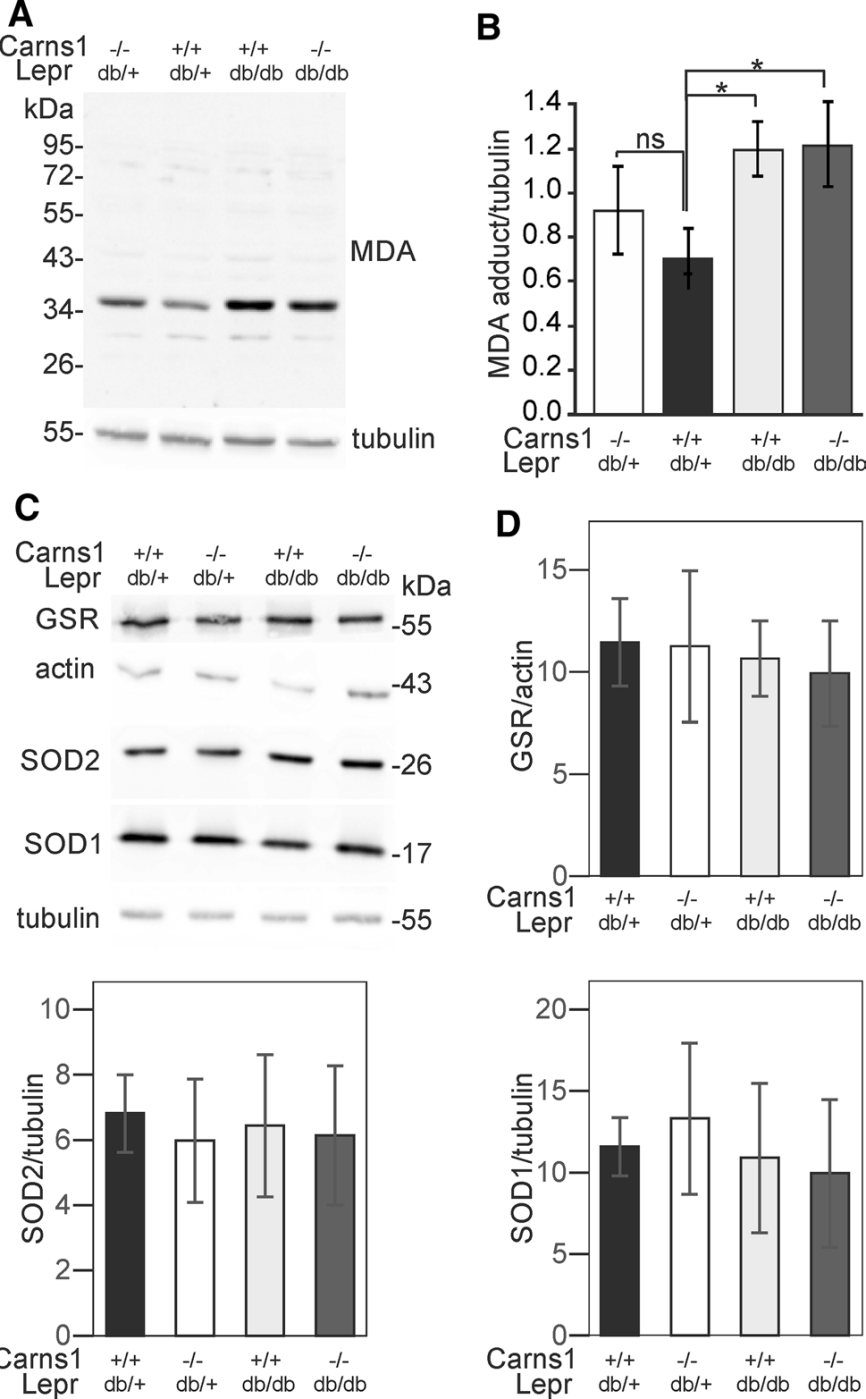
Analysis of MDA-adducts and anti-oxidant enzymes in Lepr^db/db^/Carns1^−/−^ mice. **A** Western blot analysis (10% SDS-PAGE, 20 µg protein/lane) of MDA adducts in kidneys from Lepr^db/db^/Carns1^−/−^ mice. **B** Densitometric quantification of MDA Western blots (mean ± SD, *n* = 4 mice per genotype). *p < 0.05; ns, not significant. **C** Western blot analysis (12.5% SDS-PAGE; 20 µg/lane) of anti-oxidant enzymes GSR, SOD1 and SOD2 in kidneys from 5-month-old mice (genotypes as indicated). **D** Densitometric quantification of GSR, SOD1 and SOD2 (mean ± SD, *n* = 4 mice per genotype). No significant differences were observed

Because an anti-lipoxidation effect of carnosine might be detectable only under pathological conditions associated with increased oxidative stress, we examined a diabetes mouse model (Lepr^db/db^) that is known to suffer from elevated oxidative stress and was expected to have higher levels of MDA in kidney, as suggested by higher levels of thiobarbituric acid reactive substances (DeRubertis et al. 2004). In line with this, MDA-adduct Western blot analysis revealed stronger signals in kidney extracts from 5-month-old Lepr^db/db^ mice (Fig. 5A, B) compared to controls. In agreement with the experiments shown above (Fig. 3A, B), MDA-adducts were not increased in Carns1^−/−^ mice compared to controls (Fig. 5A, B). Moreover, Carns1^−/−^/Lepr^db/db^ mice did not show a further increase in MDA-adduct levels compared to Carns1^+/+^/Lepr^db/db^ mice (Fig. 5B). Western blot analysis of GSR, SOD1, and SOD2 did not show significant differences between genotypes (Fig. 5C, D).

### Absence of carnosine does not affect body weight, blood glucose and insulin level in Lepr^db/db^ mice

**Fig. 6:**
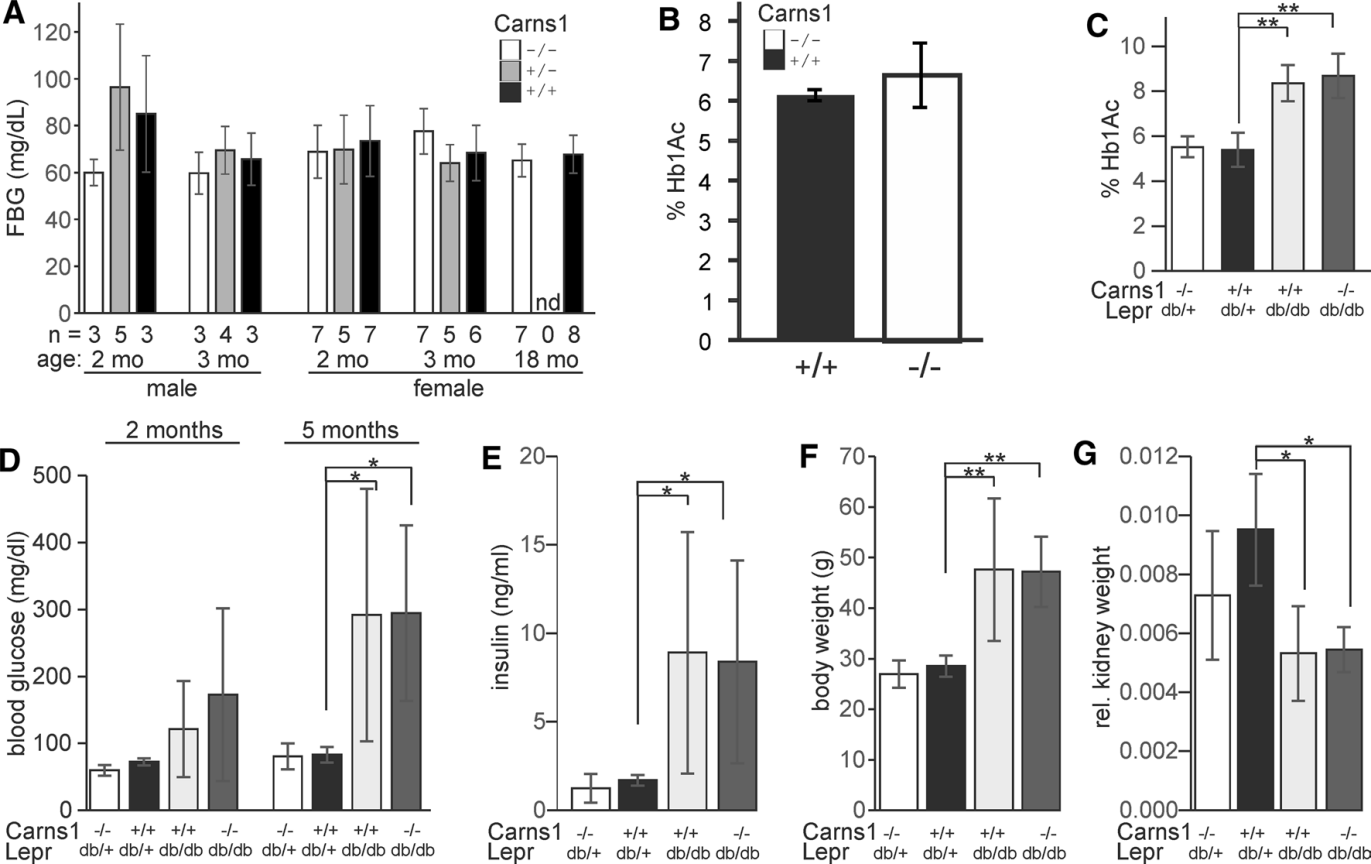
FBG, insulin and Hb1Ac levels in Lepr^db/db^/Carns1^−/−^ mice. **A** FBG was measured in male and female Carns1^−/−^, Carns1^±^ and Carns1^+/+^ mice at 2, 3 and 18 months of age (*n* = number of animals examined). No significant differences were observed. **B** Hb1Ac levels in blood from 18-month-old female Carns1^+/+^ and Carns1^−/−^ mice (*n* = 5 per genotype). No significant difference was found. **C** Hb1Ac level in blood from 5-month-old Carns1^+/+^ and Carns1^−/−^ mice (*n* = 4 per genotype). **D** FBG level in 2-month- and 5-month-old mice of the genotypes indicated. For the non-diabetic mice data from Carns1^−/−^/Lepr^db/+^ and Carns1^−/−^/Lepr^+/+^ (2 months: *n* = 11; 5 months: *n* = 8), as well as Carns1^+/+^/Lepr^db/+^ and Carns1^+/+^/Lepr^+/+^ (2 months: *n* = 24; 5 months: *n* = 14) were combined (*n* = 6 for the other genotypes). **E** Insulin concentration in 5-month-old mice of the genotypes indicated (*n* = 8 for Carns1^−/−^/Lepr^db/+^ and Carns1^+/+^/Lepr^db/+^; *n* = 6 for Carns1^−/−^/Lepr^db/+^ and Carns1^+/+^/Lepr^db/+^. **F** Body weights at 5 months of age (*n* = 6 per genotype). **G** Relative kidney weights at 5 months of age (*n* = 6 per genotype). All data shown are the mean ± SD. Data were analysed by one-way ANOVA (except **B**: *t* test): *p < 0.05, **p < 0.001 (one-way ANOVA with post hoc Tukey HSD test)

It has been shown that lower serum carnosine concentration in CN1 carnosinase-transgenic Lepr^db/db^ mice correlates with higher fasting blood glucose (FBG) level (Sauerhöfer et al. 2007). No significant difference in FBG level was observed between Carns1^−/−^ and Carns1^+/+^ mice (Fig. 6A). Glycated haemoglobin (Hb1Ac) concentration was also normal in Carns1^−/−^ mice (Fig. 6B). As expected, Hb1Ac was increased in Lepr^db/db^/Carns1^+/+^ mice, but there was no further increase in Lepr^db/db^/Carns1^−/−^ mice (Fig. 6C). We did not observe significant differences in FBG concentration in Lepr^db/db^/Carns1^−/−^ compared to Lepr^db/db^/Carns1^+/+^ mice (Fig. 6D). The two genotypes did also not differ significantly with respect to insulin plasma concentration (Fig. 6E). Relative kidney weights of Lepr^db/db^ mice were significantly reduced, but only as a consequence of the higher body weight (Fig. 6F, G). Neither absolute (not shown) nor relative kidney weights (Fig. 6G) differed significantly between Lepr^db/db^/Carns1^+/+^ and Lepr^db/db^/Carns1^−/−^ mice. This is in line with results from Sauerhöfer et al. (2007) showing that renal hypertrophy in Lepr^db/db^ mice does not correlate with carnosine concentration. In contrast to CN1-transgenic Lepr^db/db^ mice (Sauerhöfer et al. 2007), Lepr^db/db^/Carns1^−/−^ mice older than 15 weeks did not show progressive weight loss and body weights of 5-month-old Lepr^db/db^/Carns1^+/+^ and Lepr^db/db^/Carns1^−/−^ mice were not significantly different (Fig. 6F). In summary, we found no evidence that Carns1 deficiency aggravates the phenotype of Lepr^db/db^ mice.

## Discussion

Several studies, reviewed by Boldyrev et al. (2013), have demonstrated that carnosine can reduce oxidative stress in vitro and in vivo. The anti-oxidant activity of carnosine is based on its reaction with superoxide anions (Kohen et al. 1991; Klebanov et al. 1997), reactive aldehydes like HNE and acrolein (Aldini et al. 2002; Baba et al. 2013; Zhao et al. 2019; Spaas et al. 2021) or carbonyl groups in oxidized proteins (Hipkiss et al. 2001). Carnosine may also increase activity of anti-oxidant enzymes, such as superoxide dismutase or glutathione peroxidase (Kim et al. 2011). Most studies have been performed by the administration of exogenous carnosine, which leaves the role of endogenous synthesized carnosine unresolved. In part this point has been addressed using transgenic serum carnosinase CN1 overexpression to reduce carnosine concentration (Sauerhöfer et al. 2007; Everaert et al. 2020; Qiu et al. 2020) and more recently mice and rats deficient in Carns1 (Wang-Eckhardt et al. 2020; de Souza Gonçalves et al. 2021) or GADL1, an enzyme involved in the synthesis of the carnosine precursor β-alanine (Mahootchi et al. 2020). While it can be assumed that Carns1^−/−^ rodents lack HCDs in all tissues, CN1-transgene expression or carnosine/anserine supplementation primarily affect plasma and kidney ("circulating") HCD levels (Sauerhöfer et al. 2007; Qiu et al. 2019, 2020; Everaert et al. 2020, 2021). However, CN1-transgene expression also strongly reduced carnosine in brain (Qiu et al 2020), but not muscle (Everaert et al 2020). Thus, the use of CN1-transgene expression to explore the role of tissue carnosine/HCDs is limited.

Aging and several diseases, e.g. type 2 diabetes, are associated with elevated oxidative stress resulting in increased protein carbonyl content (Sohal et al. 1994; Tian et al. 1998; Moreno-Ulloa 2015; Hecker et al. 2018; Hauck et al. 2019). While we observed an age-dependent increase of protein carbonylation in aging mice and also elevated MDA protein adduct levels in Lepr^db/db^ mice, absence of Carns1 apparently did not reinforce oxidative stress in aging or Lepr^db/db^ mice. This finding suggests that in the analysed tissues other anti-oxidants play a more important role. Alternatively, other anti-oxidant systems may compensate loss of HCDs, though we could not find any evidence for an upregulation of SOD1 and SOD2, which are known to be induced in response to oxidative stress (Storz et al. 2005; Dell'Orco et al. 2016).

Potentially, absence of any significant effect on oxidative stress markers by Carns1-deficiency in contrast to models using carnosine supplementation or carnosinase overexpression could be a result of more efficient compensation by other systems in Carns1^−/−^ mice, where HCDs are completely missing throughout development.

Although our results appear to contradict the assumed anti-oxidant role of carnosine, they are in line with several studies on the effect of supplemented carnosine on oxidative stress markers, which showed no or only weak effects on MDA or protein carbonyl content in rodents under non-pathological conditions (Aydin et al. 2010; Ozel Turkzu et al. 2010; Kalaz et al. 2014). These and other studies, however, indicated that a significant anti-oxidant activity of supplied carnosine may be observed in animal models exhibiting elevated oxidative stress (Ozel Turkzu et al. 2010; Kalaz et al. 2014; Xie et al. 2017; and references in Boldyrev et al. 2013). This appears to be the case also for HCDs synthesized in tissues, as ischemia–reperfusion injury in heart muscle and HNE and acrolein adduct formation are significantly reduced in Carns1-transgenic mice (Zhao et al. 2020). Whether Carns1^−/−^ mice, however, are more susceptible towards increased oxidative stress has to be addressed in future studies.

Currently, there is evidence for a protective role of endogenously synthesized carnosine against oxidative stress only in the primary olfactory pathway, specifically the olfactory receptor neurons, which likely exhibit the highest carnosine concentration in rodent tissues (Kamal et al. 2009; Boldyrev et al. 2013). In Carns1^−/−^ mice, increased levels of protein carbonylation, though not lipoxidation end products MDA and HNE, were found in the olfactory bulb (Wang-Eckhardt et al. 2020). An anti-oxidant role of carnosine in the primary olfactory pathway is further supported by the elevated levels of GSR in the olfactory bulb of glutamate decarboxylase like 1 (GADL1)-deficient mice, which have strongly reduced carnosine levels in the olfactory bulb (Mahootchi et al. 2020).

In line with the present study, no increased oxidative stress was observed in cardiomyocytes from CARNS1^−/−^ rats (de Souza Gonçalves et al. 2021). In these rats, however, heart muscle showed impaired contractile function in combination with impaired calcium handling that indicated an important role of carnosine in excitation–contraction coupling, as also suggested for skeletal muscle in earlier studies (Dutka et al. 2012; Blancquaert et al. 2015).

There is an increased turnover of carnosine and increased activity of serum carnosinase CN1 in rodent models of diabetes and anti-diabetic properties of carnosine have been observed in several studies (Mong et al. 2011; Riedl et al. 2011; Ansurudeen et al. 2012; Peters et al. 2015, 2018b; Scuto et al. 2020). Type 2 diabetic patients have lower carnosine concentration in muscle (Gualano et al. 2012). Moreover, a polymorphism in the CNDP1 gene ("Mannheim allele") encoding serum carnosinase CN1 with reduced enzymatic activity is associated with a reduced risk of developing diabetic nephropathy (Janssen et al. 2005). Administration of carnosine increased insulin secretion and reduced FBG in two diabetes mouse models, Lepr^db/db^ (Sauerhöfer et al. 2007) and BTBR^ob/ob^ mice (Albrecht et al. 2017). In addition, reduced serum carnosine levels correlated with increased FBG and reduced insulin concentration in CN1-transgenic Lepr^db/db^ mice compared to non-transgenic Lepr^db/db^ mice (Sauerhöfer et al. 2007). Interestingly, CN1-transgenic Lepr^db/db^ mice showed massive glucosuria and significant weight loss at 15–20 weeks of age (Sauerhöfer et al. 2007). In contrast, we did not find significant differences in FBG, insulin and body weight in Carns1^−/−^/Lepr^db/db^ compared to Carns1^+/+^/Lepr^db/db^ mice. Thus, there is no absolute negative correlation between carnosine concentration and FBG level and insulin secretion under all experimental conditions, suggesting that insulin secretion is not directly influenced by carnosine. In line with this, carnosine at physiological concentrations was unable to stimulate insulin secretion in an insulinoma cell line (Sauerhöfer et al. 2007). In contrast to Carns1^−/−^/Lepr^db/db^ mice, skeletal muscle in CN1-transgenic Lepr^db/db^ mice can still release HCDs and high serum carnosinase activity likely leads to increased production of the hydrolysis products, β-alanine and histidine. If these differences may play a role to explain the apparently contradicting results obtained with the two animal models requires further studies.

In conclusion, we found no evidence to support the hypothesis that endogenously synthesized HCDs in mice are required to combat oxidative stress in those tissues that normally have the highest HCD concentrations (skeletal and heart muscle, brain and kidney). Thus, other anti-oxidant systems may play a more important role or can efficiently compensate loss of carnosine in mice. The possibility that endogenous HCDs may have a more important anti-oxidant role under pathological conditions associated with elevated oxidative stress remains to be examined.

## Supplementary Information

Below is the link to the electronic supplementary material.

Supplementary file1 (PDF 605 kb)

## Data Availability

Datasets generated in the current study are available from the corresponding author on reasonable request.

## Abbreviations

Carns1: Carnosine synthase 1
CN1: Carnosinase 1
CNDP1: Carnosine dipeptidase 1
DNP: 2,4-Dinitrophenol
DNPH: 2,4-Dinitrophenylhydrazine
FBG: Fasting blood glucose
GSR: Glutathione reductase
HCD: Histidine-containing dipeptide
HNE: 4-Hydroxynonenal
Lepr: Leptin receptor
MDA: Malondialdehyde
SOD1: Copper–zinc superoxide dismutase 1 (CuZnSOD)
SOD2: Manganese superoxide dismutase 2 (MnSOD)
