# Supplementary material for: Absence of endogenous carnosine synthesis does not increase protein carbonylation and advanced lipoxidation end products in brain, kidney or muscle
Source: Amino Acids. 2022 Mar 16;54(7):1013–23. doi: 10.1007/s00726-022-03150-8 (PMC9217836; doi:10.1007/s00726-022-03150-8)
Supplement: Supplementary file 1 — Supplementary file1 (PDF 605 kb) [file 726_2022_3150_MOESM1_ESM.pdf]

Supplementary Information

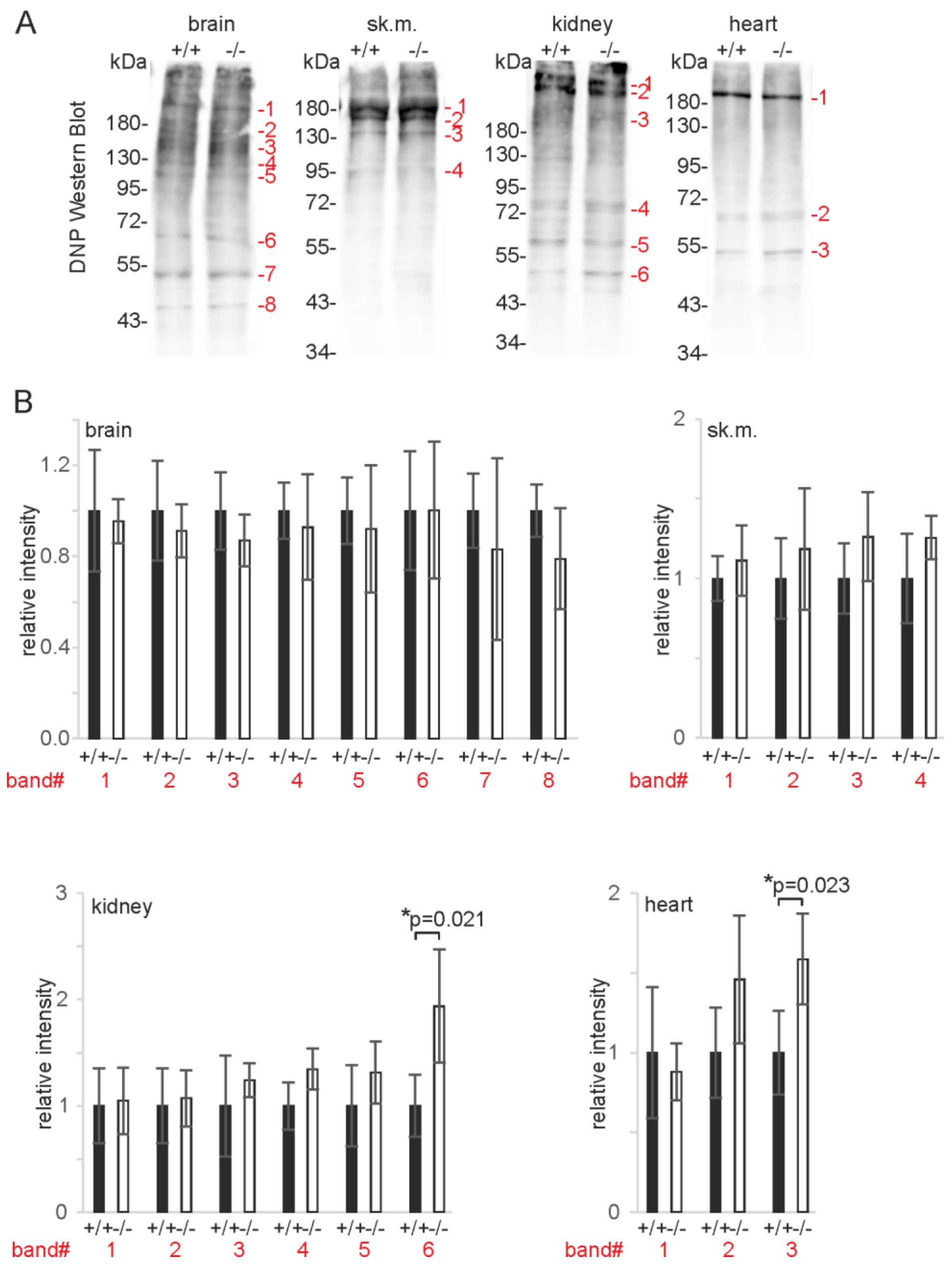

Fig. S1 Densitometric evaluation of protein carbonylation. **A** Protein carbonyl bands in DNP Western blots of Fig. 2 that were quantified individually are indicated by red numbers. **B** Densitometry of individual protein bands (or double bands, if bands are not well separated: brain #2 and #3, kidney #4). Data shown are the mean ± SD (n = 4 mice per genotype). A significant difference (increase in Carns1$^{-/-}$) was found for band #6 (50 kDa) in kidney and band #3 (53 kDa) in heart (t-test).

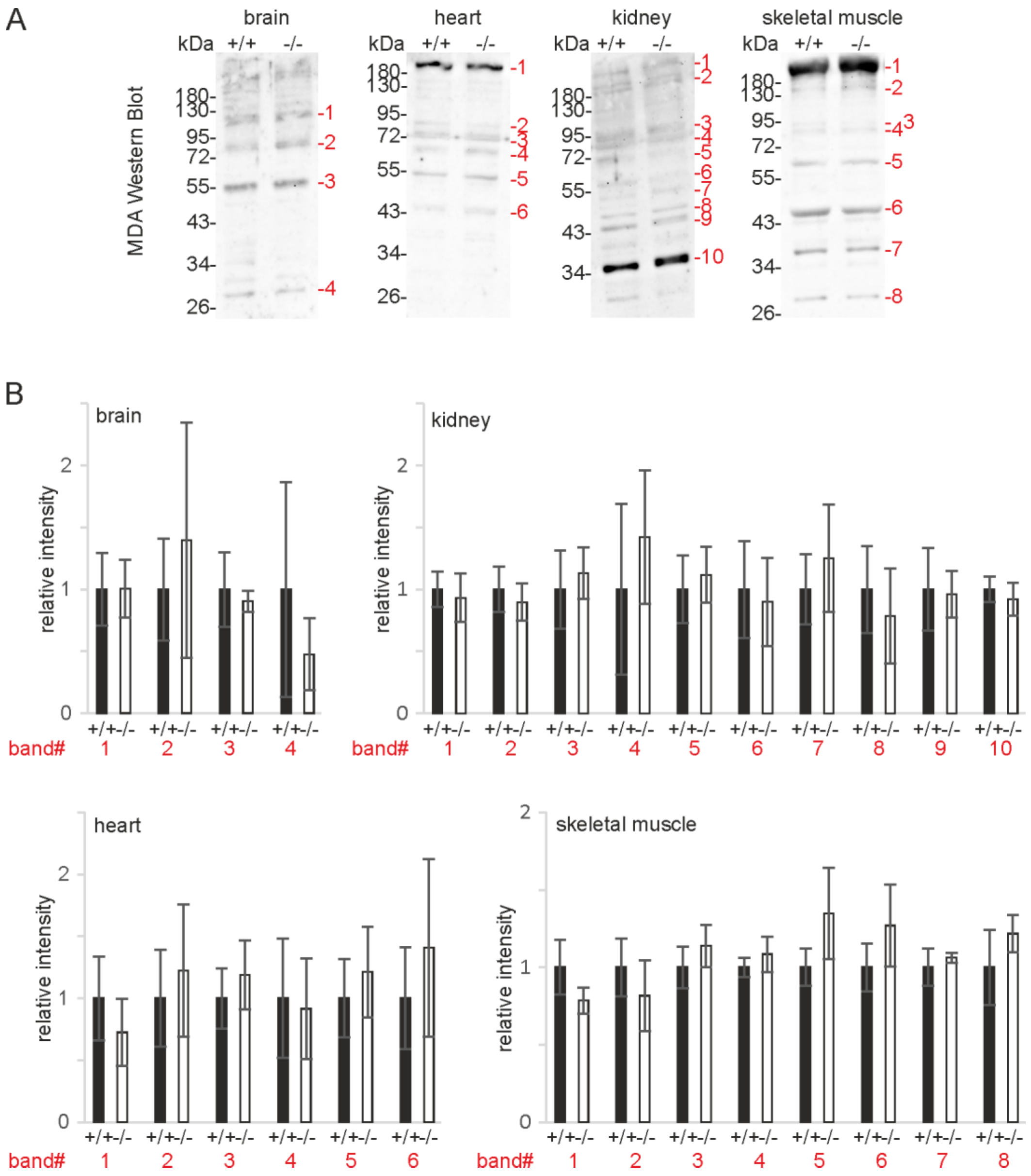

Fig. S2 Densitometric evaluation of MDA protein adducts. **A** MDA protein adduct bands of Western blots shown in Fig. 3A that were quantified individually are indicated by red numbers. **B** Densitometry of individual protein bands. Data shown are the mean ± SD (n = 4 mice per genotype). No significant differences were observed.

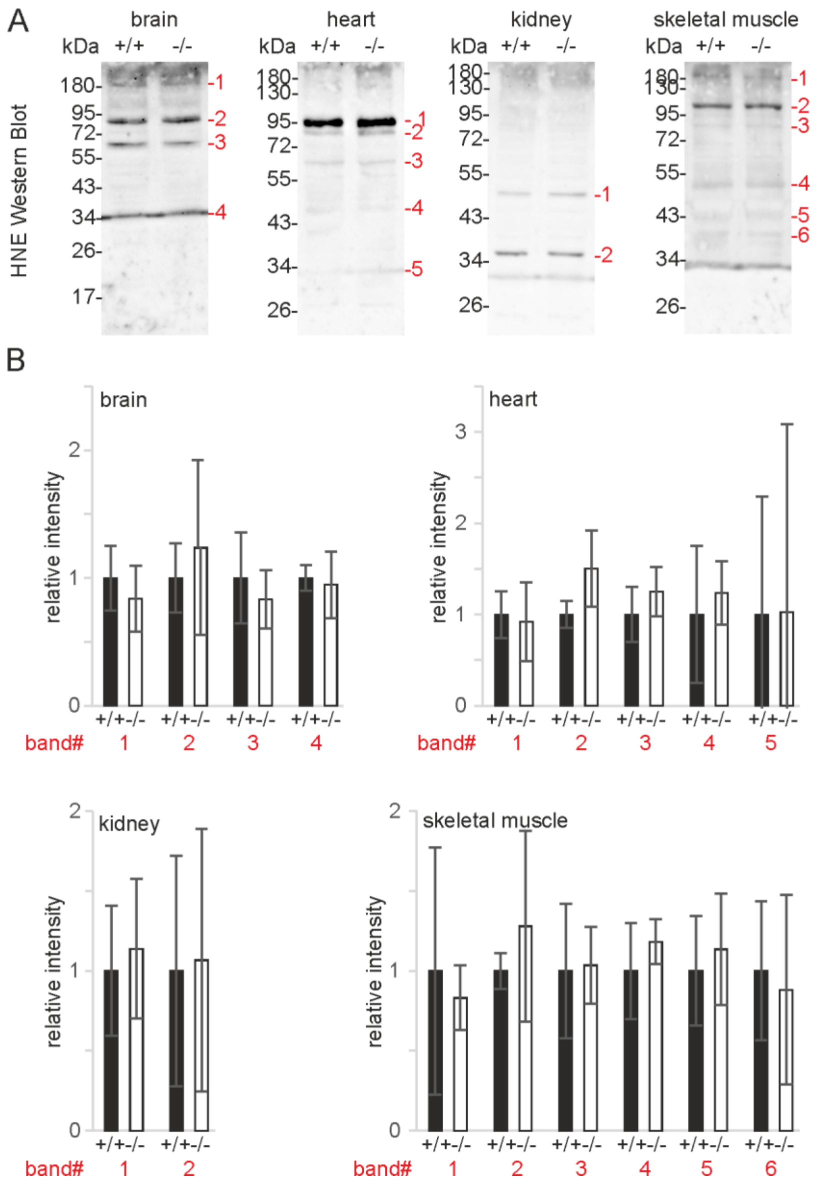

Fig. S3 Densitometric evaluation of HNE protein adducts. **A** HNE protein adduct bands of Western blots shown in Fig. 3C that were quantified individually are indicated by red numbers. **B** Densitometry of individual protein bands. Data shown are the mean ± SD (n = 4 mice per genotype). No significant differences were observed.
